# Profile of native cellulosomal proteins of *Clostridium cellulovorans* adapted to various carbon sources

**DOI:** 10.1186/2191-0855-2-37

**Published:** 2012-07-29

**Authors:** Hironobu Morisaka, Kazuma Matsui, Yohei Tatsukami, Kouichi Kuroda, Hideo Miyake, Yutaka Tamaru, Mitsuyoshi Ueda

**Affiliations:** 1 Division of Applied Life Sciences, Graduate School of Agriculture, Kyoto University, Sakyo, Kyoto, 606-8502, Japan; 2 Department of Life Science, Graduate School of Bioresources, Mie University, 1577 Kurimamachiya, Tsu, Mie, 514-8507, Japan; 3 Department of Bioinformatics, Life Science Reseach, Mie University, 1577, Kurimamachiya, Tsu, Mie, 514-8507, Japan; 4 Laboratory of Applied Biotechnology, Venture Business Laboratory, Mie University, 1577 Kurimamachiya, Tsu, Mie, 514-8507, Japan

**Keywords:** *Clostridium cellulovorans*, Cellulosome, Focused proteome analysis, Monolithic column

## Abstract

We performed a focused proteome analysis of cellulosomal proteins predicted by a genome analysis of *Clostridium cellulovorans* [Tamaru, Y., *et al..* 2010. J. Bacteriol. 192:901–902]. Our system employed a long monolithic column (300 cm), which provides better performance and higher resolution than conventional systems. Twenty-three cellulosomal proteins were, without purification, identified by direct analysis of the culture medium. Proteome analysis of the *C. cellulovorans* cellulosome after culture in various carbon sources demonstrated the production of carbon source-adapted cellulosome components.

## Introduction

The development of white biotechnology requires degradation of biomass using biocatalysts. The cellulosome, which is produced by many cellulolytic gram-positive anaerobic bacteria such as *Clostridium*, efficiently degrades plant cell wall polysaccharides. However, the molecular mechanism of cellulosome formation has not been characterized. We sequenced the entire genome of *Clostridium cellulovorans* (Tamaru et al. [2010a]) and identified all genes, including those that encode proteins of known and unknown functions, related to cellulosome composition (Tamaru et al. [2010a]). Genome analysis of *C. cellulovorans* indicated the presence of 57 cellulosomal enzymes-encoding genes including 4 scaffold proteins-encoding genes and 53 cellulosomal enzymes-encoding genes with dockerin domains (Doi and Tamaru [2001]). The major scaffold protein, CbpA, is comprised of 9 cohesin domains that bind to various cellulosomal enzymes via cohesin-dockerin interactions. Most cellulosomal enzymes are glycoside hydrolases, but they possess an interesting property. The cellulosome consists not only of glycoside hydrolases but of other proteins such as proteases, protease inhibitors, and unknown proteins. This characteristic may be important in the degradation of various resources. However, we have only general subcellular proteinous information regarding the proteins that actively degrade biomass, although genome analysis has provided many interesting insights into the characteristics of *C. cellulovorans*. The next step was to directly analyze cellulosomal proteins in the culture media.

Although proteome analysis has been advanced by the development of instruments such as the mass spectrometer (Link et al. [1999]), but difficulties remain. Ionization suppression in mass spectrometry is a significant problem that needs to be overcome for mass spectrometry to be used as a detector in proteome analysis. Results can be improved by complete pre-separation; construction of an efficient separation system is indispensable to obtain satisfactory measurement. The monolithic silica column is a novel separation medium of liquid chromatography that exhibits lower column back pressure owning by its high permeability. This property enables use of the long column that is impossible by conventional particle-packed columns and shows higher performance in comparison to conventional columns (Minakuchi et al. [1996], Minakuchi et al. [1998]). In addition, monolithic silica can be prepared in a long-fused silica capillary (300 cm) because of the attachment of the silica monolithic skeletons to the tube wall (Motokawa et al. [2002]). Proteome analysis methods could be improved by using long ultra-performance monolithic columns to overcome ionization suppression.

In this study, a novel analysis using the long monolithic column was applied to a proteome analysis focused on the cellulosome of *C. cellulovorans* to characterize the molecular mechanism that underlies efficient degradation of various biomasses.

## Materials and methods

### Cell culture and medium

*C. cellulovorans* 743B (ATCC 35296) was grown anaerobically as described (Robert et al. [1984]) except for the carbon sources, which was 0.3% (w/v) cellobiose, 0.3% (w/v) avicel, or 0.3% (w/v) xylan.

### Sample preparation of cellulosomal proteins for proteome analysis

Proteome samples were prepared from *C. cellulovorans* culture media. The culture (50 mL) was centrifuged (6,000 *g*, 25°C) and the supernatant was subjected to ultrafiltration using Amicon Ultra YM-10 (Millipore) to obtain the cellulosomal proteins (Adams et al. [2010]). The collected proteins were reduced with 10 mM tris(2-carboxyethyl)phosphine for 30 min and alkylated with 20 mM iodoacetamide for 60 min at room temperature. After acetone precipitation, the proteins were solubilized in 200 mM triethylammonium bicarbonate, trypsin-digested, and applied to a proteome analysis system.

### Protein identification of cellulosomal proteins

Protein identification was performed by a liquid chromatography/mass spectrometry system. Proteolytic digests were separated by reversed-phase chromatography using a Prominence nano flow system (Shimadzu). A monolithic silica capillary column, prepared from a mixture of tetramethoxysilane and methyltrimethoxysilane (300 cm long, 0.1 mm ID) as described in (Motokawa et al. [2002]), was used at a flow rate of 500 nL/min. The gradient was provided by changing the mixing ratio of the 2 eluents; A, 0.1% (v/v) formic acid, and B, acetonitrile containing 0.1% (v/v) formic acid. The gradient was started with 5% B, increased to 45% B for 600 min, further increased to 95% B to wash the column, then returned to the initial condition, and held for re-equilibration. A packed tip column (NTCC-360, 150 mm × 100 μm I.D., Nikyo technos, Tokyo) was used as conventional packed column at a flow rate of 500 nL/min in gradient time 60 min. The separated analytes were detected on an LTQ Velos linear ion trap mass spectrometer (Thermo Scientific). For data-dependent acquisition, the method was set to automatically analyze the top 3 most intense ions observed in the MS scan. An ESI voltage of 2.4 kV was applied directly to the LC buffer distal to the chromatography column using a microtee. The ion transfer tube temperature on the LTQ Velos ion trap was set to 300°C. The mass spectrometry data were used for protein identification by Protein Discoverer software (Thermo Scientific) with the protein database built from genome analysis of *C. cellulovorans* (Tamaru et al. [2010a]). The data were then filtered at a q-value ≤ 0.01 corresponding to 1% FDR on a spectral level.

## Results

### Construction of proteome analysis system using a long monolithic column

**Figure 1:**
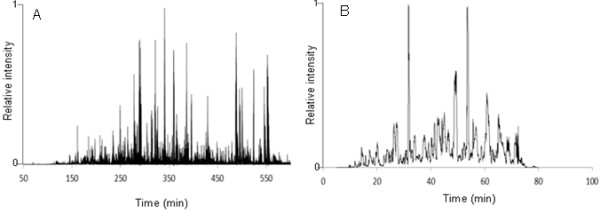
**Base peak chromatogram of a proteome sample extracted from*C. cellulovorans*(A) monolithic column (300 cm), (B) conventional packed column (15 cm).** A proteome sample prepared from *C. cellulovorans* was injected onto the column, and a (A) 600 or (B) 60 min gradient was applied with a flow rate of 500 nL/min.

By base peak chromatograms of the same trypsin-digested proteome sample, which is digested from cellulosomal proteins in culture medium by trypsin, comparison of separation by a long monolithic column (300 cm) and a conventional packed column (15 cm) were shown in Figure1. A peak capacity of the long monolithic column is estimated to be ca. 300, compared with that of the conventional column (ca. 50). With the monolithic column, we identified 679 non-redundant peptides from 193 proteins while the conventional column yielded 46 peptides from 26 proteins. In the identification of the scaffold protein CbpA, 26% of the sequence coverage, including some peptides that could not be detected using the conventional column by which only 2% of the sequence coverage was identified, was performed by the monolithic column.

### Protein identification of cellulosomal proteins cultured in various carbon sources

Proteome samples prepared from cells cultivated as described previously (Robert et al. [1984]), with 0.3% cellobiose, 0.3% avicel, or 0.3% xylan, were analyzed by the new system using the long monolithic column to characterize protein production. The numbers of identified cellulosomal proteins were summarized in Tables 1 and 2. 

**Table 1:** Identified cellulosomal proteins by proteome analysis

**Functions**		**Gene name**	**CAZy**^**a**^	**Carbon sources**			**Accesion No**^**b**^
				**Cellobiose**	**Avicel**	**Xylan**	
Cellulases							
	endoglucanase		GH5			○	ZP_04806172
	endoglucanase		GH9	●	●	●	ZP_04806149
	endoglucanase		GH5		○	○	ZP_04806690
	endoglucanase		GH5			○	ZP_04804560
	endoglucanase		GH5	●	●	●	ZP_04804999
	endoglucanase	*EngE*	GH5	●	●	●	AAD39739
	endoglucanase	*EngH*	GH9			○	ZP_04807564
	endoglucanase	*EngK*	GH9			○	ZP_04807563
	endoglucanase	*EngL*	GH9	●	●	●	ZP_04807561
	endoglucanase	*EngY*	GH9	●	●	●	ZP_04804221
Hemicellulases							
	mannanase	*ManA*	GH5	●	●	●	ZP_04807560
	mannanase		GH26	●	●	●	YP_003845544
	mannanase		GH26	●	●	●	ZP_04805612
	mannanase		GH26	●	●	●	ZP_04806148
	xylanase	*XynA*	GH11			○	ZP_04805534
	xylanase	*XynB*	GH10		○	○	ZP_04807887
	exocellulase	*ExgS*	GH48	●	●	●	AAC38571
Pectate lyases							
	pectate lyase		PL1		○	○	YP_003842527
	pectate lyase	*PelA*	PL9		○	○	AAG59609
Other proteins							
	peptidase inhibitor				○	○	ZP_04807292
	peptidase inhibitor				○		ZP_04807290
	peptidase					○	ZP_04804668
	sialicacid-specific 9-O-acetylesterase				○	○	ZP_04805106
	hypothetical protein				○		ZP_04804379
	hypothetical protein			●	●	●	YP_003843744
Scaffold proteins							
	cellulose binding protein	*CbpA*		●	●	●	AAA23218
	hydrophobic protein	*HbpA*			○		AAF06108

^a^ Refer to http://www.cazy.org/.

^b^ Refer to http://www.ncbi.nlm.nih.gov/.

● (closed circles): Common proteins identified in each substrate.

○ (open circles): Not common proteins identified in each substrate.

**Table 2:** Comparison of the number of identified cellulosomal enzymes

		**Genome analysis**^**a**^	**Carbon sources**			
			**Cellobiose**	**Avicel**	**Xylan**	**All substrates**^**b**^
**Cellulosomal enzymes**						
	Celllulases	16	5	6	10	5
	Hemicellulases	11	5	6	7	5
	Pectate lyases	2	0	2	2	0
	Other proteins	24	1	5	4	1
	total	53	11	19	23	11

^a^ Enzymes categorized in the reference (Tamaru et al. [2010b]).

^b^ The number of common enzymes identified in each substrate, based on Table1.

## Discussion

The quality of the mass spectrum is very important in proteome analysis. The number of identified proteins is dependent on the number of detected peptides, which is dependent on the efficiency of the separation prior to ionization. Thus, the separation performance of the liquid chromatography method directly influences the quality of the proteome analysis. De Godoy et al ([2008]) reported that complete pre-separation contributed quality of proteome analysis such as the number of identified proteins. We examined the validity of a proteome analysis system using a long monolithic column (300 cm) which has higher resolution versus a conventional packed column (15 cm). The long monolithic column showed good separation and decreased ionization suppression. In the proteome analysis with the conventional system, protein purification was required because the number of detectable peptides was reduced by ionization suppression. With the new system, proteome analysis of cellulosomal proteins could be performed directly from crude extracted samples thanks to the ultra-performance separation by the long monolithic column. By this benefit, the analysis of non-cellulosomal enzyme of *C. cellulovorans* became possible simultaneously. The ratio of cellulosomal / non-cellulosomal genes encoding glycosyl hydrolases and polysaccharide lyases of *C. cellulovorans* was 0.48 (29/61) while that of *C. thermocellum* which is other cellulosome-producing clostridia was 3.31(53/16) (Tamaru et al. [2010b]). From proteome analysis of supernatants, total 59 proteins annotated glycosyl hydrolases and polysaccharide lyases were identified and its ratio of cellulosomal / non-cellulosomal was 0.69 (24/35). It was confirmed by proteome analysis that *C. cellulovorans* has much non-cellulosomal enzyme as compared with other clostridia. The degradation of various resources by *C. cellulovorans* is highly dependent on not only cellulosomal enzymes but also these secretory proteins.

To analyze the mechanism of cellulosome formation, our proteome analysis focused on the cellulosomes of *C. cellulovorans*. Although 57 cellulosomal genes including 4 scaffold proteins and 53 cellulosomal enzymes were identified by a genome analysis of *C. cellulovorans* (Tamaru et al. [2010b]), what kinds of cellulosomal proteins have been produced in various carbon sources has not been completely confirmed (Oded and Doi [1990]).

**Figure 2:**
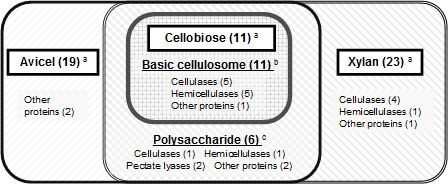
**Classification of produced cellulosomal enzymes of*C. cellulovorans*cultivated with the various carbon sources, based on Table**1**.**^a^ The number in the panel indicates identified enzymes cultivated with each carbon source. ^b^ The number indicates common enzymes identified in indicated carbon source. ^c^ The number indicates common enzymes identified in polysaccharides (common to avicel and xylan) but not in cellobiose.

From our experiments, the number and members of cellulosomal enzymes were found to be changeable altered, caused by the benefit of good separation using the better performance system equipped with a long monolithic column (300 cm). This suggests that *C. cellulovorans* may change cellulosomal components to match its carbon sources. In addition, a set of 11 enzymes described as closed circles in Table1 including 5-assingned cellulases, 5-assingned hemicellulases, and 1-assingned other protein identified in all culture conditions were the same enzymes produced by cultivation with cellobiose (disaccharide made from units of glucose), which is the simplest substrate. We hypothesized that a basic cellulosome could be constructed by this set of 11 enzymes. Cellulosomes have been formed by adding other cellulosomal enzymes described as open circles in Table1 necessary for degradation of polysaccharides such as avicel (polysaccharides made from units of glucose) and xylan (polysaccharides made from units of xylose) (Figure2). It is interesting to note that these adding functions of enzymes included many other proteins (Table2), whose functions in saccharification, have not been clarified. The functions of these proteins categorized into ‘other proteins’ have been not made clear in the previous reports on the analyses of natural cellulosomal proteins, although cellulosomal protease/peptidase inhibitors (named cyspins) of *C. cellulovorans* classified into other proteins showed inhibition activities by heterologous gene expression systems (Meguro et al [2011]). These other proteins might play some an important role in the efficient degradation of each biomass. We will further investigate the stoichiometric ratio of cellulosomal enzymes and proteins in different cultivation media. From these results, we hope to develop a model for the formation of cellulosomes by the change of carbon sources and to apply it to utilize the various waste biomasses. 

## Competing interests

The authors declare that they have no competing interests.
